# Editorial: Retinal Immunobiology and Retinopathy

**DOI:** 10.3389/fimmu.2021.758375

**Published:** 2021-09-01

**Authors:** Darren J. Lee, Heping Xu, Andrew W. Taylor

**Affiliations:** ^1^Department of Microbiology and Immunology, University of Oklahoma Health Sciences Center, Oklahoma City, OK, United States; ^2^Department of Ophthalmology/Dean McGee Eye Institute, University of Oklahoma Health Sciences Center, Oklahoma City, OK, United States; ^3^The Wellcome-Wolfson Institute for Experimental Medicine, Queen’s University Belfast, Belfast, United Kingdom; ^4^Department of Ophthalmology, Boston University School of Medicine, Boston, MA, United States

**Keywords:** ocular immune privilege, age-related macular degeneration, diabetic retinopathy, glaucoma, uveitis, immune tolerance, retinal pigment epithelial cells, microglial cells

The term “Ocular Immune Privilege” (OIP) was used by Sir Peter Medawar in 1948 to describe the observation that a foreign tissue graft placed in the anterior chamber of the eye did not undergo rejection (1). In the last 72 years advances in the understanding of the mechanisms that contribute to the maintenance of OIP have been made (2). These mechanisms include: 1) sequestration, a lack of lymphatics, and tight junctions that create an efficient blood-barrier surrounding retinal vessels lining the inner retina, and tight junctions between retinal epithelial cells separating the outer retina from the highly vascular choroid; 2) expression of inhibitory soluble and membrane bound factors, such as transforming growth factor-beta, alpha-melanocyte stimulating hormone, FasL, and thrombospondin; and 3) inhibition of systemic immune responses, such as the induction of regulatory T cells and suppressive antigen presenting cells. While the benefit of OIP is the protection of delicate intraocular tissues from inflammation-mediated damage, it leaves the eye vulnerable to infection. Furthermore, while the mechanisms of OIP are considerable, diseases such as autoimmune uveitis, diabetic retinopathy, age-related macular degeneration, and optic neuropathy can break through the mechanisms of OIP. We are pleased to present a compendium of original research and review articles on the Research Topic “Retinal Immunobiology and Retinopathy” that discuss the mechanisms of OIP and the diseases of interest.

A thorough discussion of OIP mechanisms and the treatments available for retinal disease is reviewed by Takeda et al. This review provides a detailed discussion of immunological mechanisms that contribute to non-infectious or autoimmune uveitis, diabetic retinopathy, retinopathy of prematurity, retinitis pigmentosa, and vitreoretinal lymphoma.

Microglia cells are important in maintaining OIP and are present throughout the retina as reviewed by Rashid et al. In this timely and broadly relevant review, the origin and maintenance of retinal microglia, the role of microglia in the healthy and diseased retina, and therapies for retinal diseases that target microglia are discussed. Since microglia are present throughout the retina, they can further modulate the immune response by augmenting or inhibiting ocular inflammation. Therefore, a greater understanding of the role that microglial cells have in ocular inflammation could come from knowing the transcriptional signature of microglia during inflammation. As such, Bell et al. have characterized the transcriptome of microglia during the “early” activation, peak, and two weeks after endotoxin induced uveitis (EIU) and found that the acute transcriptional changes that occur to microglia during EIU return to their original state. The EIU model shows that the activation of endogenous inflammatory pathways can override the mechanisms of OIP. Mursalin et al. describe the resulting rapid and disastrous inflammation that can happen following Toll-like receptor (TLR)-2 and TLR-4 stimulation within the eye.

Age-related macular degeneration (AMD) is the largest cause of blindness resulting from photoreceptor degeneration. Myeloid derived suppressor cells (MDSCs) are suppressive immune cells from the myeloid lineage initially identified by their association with tumor cells for their potent immunosuppressive capacity. The role of MDSCs in the maintenance of OIP to prevent AMD is reviewed by Kauppinen et al. In fact, CX3CR1^-/-^ mice exhibit an increased number of mononuclear phagocytes in the subretinal space and is associated with photoreceptor degeneration. Lavalette et al. show that CD36, a member of the class B scavenger receptor family involved in TLR-signaling on mononuclear phagocytes can promote sterile inflammation, and is necessary for photoreceptor degeneration in two different CX3CR1^-/-^ AMD models. An important component of OIP is maintenance of the blood ocular barrier. An elegant study by Matsubara et al. describes how AMD results from loss of barrier function through secretion of granzyme B.

Diabetic retinopathy is the most common microvascular complication of diabetes, and is the largest cause of blindness among those of working-age. Inflammation and angiogenesis are hallmarks of diabetic retinopathy. The proangiogenic and proinflammatory cytokine macrophage migration inhibitory factor (MIF1) is an upstream activator of innate immunity. El-Asrar et al. show that MIF1 is significantly elevated in proliferative diabetic retinopathy (PDR), illustrating how diabetes diminishes OIP. Another component of inflammation is the complement system. Apart from C1q for the purpose of synaptic pruning, complement activation does not normally occur inside the eye, but has been observed in the diabetic retina. Shahulhameed et al. report a significant elevation of C3 deposition in the capillary wall and the co-localization of Complement Factor H in activated CD11b^+^ microglia in retina samples from diabetic donors. This suggests a feedback mechanism for arresting excessive complement activation in the diabetic retina by activated microglia.

Uveitis is inflammation of the eye and the third leading cause of blindness in countries with healthcare access. The majority of chronic uveitis cases are idiopathic or can be associated with a systemic autoimmune disease. As such, targeting inhibition of inflammatory pathways can be an effective treatment for uveitis. Efforts are underway to identify normally expressed signaling molecules that suppress the immune response, such as kallistatin, a serine proteinase inhibitor with anti-inflammatory properties. A report by Muhammad et al. demonstrates the suppression of experimental autoimmune uveitis (EAU) with kallistatin, and further shows that kallistatin induces regulatory T cells. T cell function is modulated through epigenetic modification, and two inhibitors that block DNA methylation have been approved for human disease. Zou et al. demonstrate that the DNA methylation inhibitor, zebularine, is effective in suppressing inflammatory T cells, promoting regulatory T cells, and suppresses EAU.

Glaucoma is the most prevalent neurodegenerative disease marked by progressive damage to the optic nerve and death of retinal ganglion cells. Therapies for glaucoma are based on lowering the intraocular pressure (IOP), but because elevated IOP is not necessary or sufficient to cause glaucoma-related neuropathy, there are likely additional mechanisms involved. In the mini-review by Jiang et al., they discuss the evidence that the neuropathy occurs due to a loss of OIP resulting in T cell-related low-grade intraocular inflammation.

The 12 featured articles in this Research Topic: Retinal Immunobiology and Retinopathy provide a broad discussion of the mechanisms of OIP, demonstrate that in different retinal diseases inflammatory pathways can break through the mechanisms of OIP, and show that therapies that have modes-of-action like the mechanisms of OIP are effective at treating retinal disease. While many therapies to treat retinal inflammatory diseases have been developed, additional mechanisms remain to be elucidated to develop better and more effective therapies for retinal inflammatory diseases.

## Author Contributions

The writing of this editorial was a collaborative effort between DL, HX, and AT. DL wrote the first draft which was then revised by HX and AT. All authors contributed to the article and approved the submitted version.

## Funding

Grant funding: EY024951 (DL) and EY025961 (AT).

## Conflict of Interest

AT has a sponsored research agreement and is a consultant with Palatin Technologies Inc. (Cranbury, New Jersey). HX has a sponsored research agreement with Boehringer Ingelheim and a consultant agreement with F. Hoffmann-La Roche Ltd.

The remaining author declares that the research was conducted in the absence of any commercial or financial relationships that could be construed as a potential conflict of interest.

## Publisher’s Note

All claims expressed in this article are solely those of the authors and do not necessarily represent those of their affiliated organizations, or those of the publisher, the editors and the reviewers. Any product that may be evaluated in this article, or claim that may be made by its manufacturer, is not guaranteed or endorsed by the publisher.
